# An Autonomic Neuroprosthesis: Noninvasive Electrical Spinal Cord Stimulation Restores Autonomic Cardiovascular Function in Individuals with Spinal Cord Injury

**DOI:** 10.1089/neu.2017.5082

**Published:** 2018-02-01

**Authors:** Aaron A. Phillips, Jordan W. Squair, Dimitry G. Sayenko, V. Reggie Edgerton, Yury Gerasimenko, Andrei V. Krassioukov

**Affiliations:** ^1^ ICORD-BSCC, University of British Columbia, Vancouver, British Columbia, Canada.; ^2^ Department of Integrative Biology and Physiology, University of California, Los Angeles, Los Angeles, California.; ^3^ Neurobiology, University of California, Los Angeles, Los Angeles, California.; ^4^ Department of Neurosurgery, David Geffen School of Medicine, University of California, Los Angeles, Los Angeles, California.; ^5^ Brain Research Institute, University of California, Los Angeles, Los Angeles, California.; ^6^ Pavlov Institute of Physiology, Saint-Petersburg, Russia.

**Keywords:** autonomic, cardiovascular, neurological injury, spinal cord, spinal cord injury, stimulation

## Abstract

Despite autonomic dysfunction after spinal cord injury (SCI) being the major cause of death and a top health priority, the clinical management options for these conditions are limited to drugs with delayed onset and nonpharmacological interventions with equivocal effectiveness. We tested the capacity of electrical stimulation, applied transcutaneously over the spinal cord, to manage autonomic dysfunction in the form of orthostatic hypotension after SCI. We assessed beat-by-beat blood pressure (BP), stroke volume, and cardiac contractility (dP/dt; Finometer), as well as cerebral blood flow (transcranial Doppler) in 5 individuals with motor-complete SCI (4 cervical, 1 thoracic) during an orthostatic challenge with and without transcutaneous electrical stimulation applied at the TVII level. During the orthostatic challenge, all individuals experienced hypotension characterized by a 37 ± 4 mm Hg decrease in systolic BP, a 52 ± 10% reduction in cardiac contractility, and a 23 ± 6% reduction in cerebral blood flow (all *p* < 0.05), along with severe self-reported symptoms. Electrical stimulation completely normalized BP, cardiac contractility, cerebral blood flow, and abrogated all symptoms. Noninvasive transcutaneous electrical spinal cord stimulation may be a viable therapy for restoring autonomic cardiovascular control after SCI.

## Introduction

Over the past 10 years, we have come to realize that electrical stimulation of the spinal cord caudal to the injury has the capacity to both directly excite spinal cord neurons and also modulate spinal circuity.^1–6^ Although the recovery of walking has been the focus of the vast majority of research into electrical stimulation after spinal cord injury (SCI), the recovery of normal autonomic cardiovascular function is consistently reported to be more urgently desired by people living with SCI.^7^ Based on the present understanding of somatovisceral integration within the spinal cord, it is plausible that electrical spinal cord stimulation can acutely excite and/or modulate spinal autonomic circuits to rapidly normalize cardiovascular function after SCI.^7^ In support of this contention, some early work in uninjured individuals have observed that electrical spinal stimulation can elicit acute cardiovascular effects, including altered blood flow and potentially cardiac function.^8–10^

Autonomic dysfunction permeates into all aspects of life after SCI, with particularly insidious cardiovascular consequences.^7^ Orthostatic hypotension (OH) is a major concern after SCI and is defined as a 20 mm Hg or more decrease in systolic blood pressure (BP).^7^ The majority of those living with SCI experience OH, which often leads to the loss of consciousness,^7^ and has been shown to result in impaired cognitive function.^7,11^ Over the long term, repetitive exposure to OH is associated with declining cardiovascular/cerebrovascular health^12–14^ and likely underlies the 3- to 4-fold elevated odds and risk of cardiovascular and cerebrovascular diseases after SCI, which are the primary causes of death.^15,16^ Pharmacological and nonpharmacological options for managing OH are limited. Alpha-agonist administration 1 h before an episode of OH is capable of acutely improving BP as well as integrated cardiac and cerebrovascular functions.^7,11,17,18^ Although pharmacological approaches certainly have a role clinically, they have significant limitations given that they require a number of minutes/hours to become active, and then persist for hours, with significant side effects.^19^ Conversely, OH experienced by people with SCI is often unexpected and much more transient, occurring in just minutes.^7^ One recent study focused on restoring lower-limb function through electrical spinal cord stimulation mentioned anecdotes indicating that autonomic function may be acutely improved in their spinal cord–injured participants, but did not provide any supporting data.^5^ No study has evaluated the potential for electrical spinal cord stimulation to restore autonomic cardiovascular function after SCI.

Herein, we introduce a novel strategy to manage a cardiovascular dysfunction system using noninvasive transcutaneous electrical spinal cord stimulation after SCI. We reasoned that stimulation of the thoracic spinal cord, where sympathetic preganglionic neurons cell bodies are located, would elicit acute increases in BP, cerebral blood flow, and cardiac function when they were dangerously reduced because of orthostasis.

## Methods

Four individuals all sustained motor-complete cervical injury (C5 American Spinal Injury Association Impairment Scale [AIS]-B; C5 AIS-B; C5 AIS-A; C6 AIS-A) whereas the fifth was motor complete at the T2 level AIS-A (1 female), all occurring at least 3 years before the assessments. All participants were between 23 and 32 years of age and instructed to prepare for the testing day as previously described,^20^ and provided written informed consent in accord with the University of California, Los Angeles (Los Angeles, CA) Institutional Review Board, who approved this study. Participants were tested over a 1-h protocol that consisted of at least 15 min of resting in the supine position, while being outfitted with assessment equipment, followed by at least 10 min of supine rest, which preceded a progressive orthostatic challenge that has been previously described and shown to be a reliable test of OH.^21^

Participants were asked to rank their symptoms of nausea/dizziness 1–10 (10 being most severe) each minute of the test. After BP decreased sufficiently to clinically indicate OH (i.e., a 20 mm Hg decrease in systolic BP), transcutaneous stimulation was applied using a self-adhesive electrode with a diameter of 30 mm (ValueTrode) placed on the skin between TVII and TVIII spinous processes (approximately corresponding to the T8 spinal segment) at the midline over the vertebral column as a cathode, and two 5 × 9 cm self-adhesive electrodes (ValueTrode) located symmetrically on the skin over the iliac crests as anodes. The stimulation was delivered at 30 Hz as monophasic, 1-ms pulses, to provide afferent input to the region of the spinal cord where sympathetic preganglionic neuron cell bodies are located.^7^ The current was increased from 10 mA until BP was normalized, up to a maximum 70 mA, and maintained for at least 1 min. Electromyography (EMG) of the lower-limb skeletal muscles was also recorded to confirm that skeletal muscle contractions were not occurring and therefore the pressor responses were not attributed to the skeletal muscle pump of the venous vasculature.^22^ Bipolar surface electrodes placed bilaterally on the vastus lateralis (VL), rectus femoris (RF), and medial hamstrings (MH), tibialis anterior (TA), soleus (SOL), and medial gastrocnemius (MG) muscles with fixed interelectrode distance of 20 mm. The EMG signals were differentially amplified using the PowerLab System (PowerLab; ADInstruments, Dunedin, New Zealand) with a band-pass filter of 10 Hz to 1 kHz. The EMG data were digitized at a sampling rate of 4000 Hz.

For each participant, brachial BP was measured (Dinamap, General Electric Pro 300V2; General Electric Tampa, FL) on the left brachial artery each minute throughout the protocol. Beat by beat blood flow velocity in the middle and posterior cerebral arteries (MCA, PCA; Transcranial Doppler, Spencer Technologies, Redmond, WA) and BP (Finometer PRO; Finapres Medicine Systems, Amsterdam, The Netherlands) were recorded and collected as previously described.^20^ Systolic and diastolic BP (SBP, DBP), as well as peak MCA blood velocity and PCA blood velocity (MCAv, PCAv) and minimum MCAv/PCAv, were then extracted from three time points: 1) supine rest (at least 10–15 sec of continuous resting data); 2) the three heart beats corresponding to minimum BP during the orthostatic challenge; and 3) during 10–15 sec of stable BP during transcutaneous stimulation. From these values, mean arterial pressure (MAP) as (2*DBP+SBP)/3 and mean MCAv/PCAv as (2*MCAv/PCAv minimum+MCAv/PCAv maximum)/3 were calculated. Stroke volume, cardiac output, and cardiac contractility (dP/dt) were calculated from the BP waveform with the Modelflow method, incorporating age, sex, height, and weight (BeatScope 1.0 software; TNO TPD Biomedical Instrumentation, Milan, Italy).^23^ Cardiac output (Q) was calculated as stroke volume (SV)*heart rate (HR). Total peripheral resistance (TPR) was calculated as MAP/Q. All hemodynamic variables were assessed using a one-way repeated measures analysis of variance (ANOVA) with Bonferroni contrast tests between time points (supine, orthostatic challenge, and orthostatic challenge with stimulation). Significance was set *a priori* at *p* < 0.05.

## Results

**FIG. 1.**
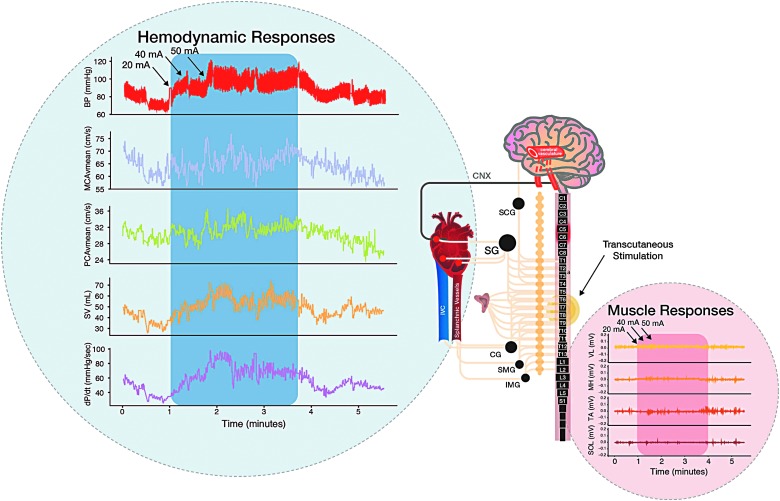
Vignette: Electrical spinal cord stimulation improved integrated cardiovascular responses to orthostatic challenge. Participant: female, 32 years of age, spinal cord injury at C6 (AIS-A), injured August 2009. Left blue inlet: Although experiencing severe orthostatic hypotension when assuming upright posture, electrical stimulation at the TVII level restored blood pressure, cerebral blood flow, cardiac function, and symptoms of orthostatic intolerance to supine levels. Note: Increasing current (20, 40, and 50 mA) resulted in step-wise increases in cardiovascular function. Right red inlet: electromyography recording of lower limbs shows that skeletal muscle contraction was not activating the skeletal muscle pump of the venous vasculature, indicating that excitation of sympathetic preganglionic neurons was responsible for the cardiovascular restoration. Note: Without stimulation, self-reported symptoms of pre-syncope were severe, being between 6 and 9, whereas with stimulation symptoms were completely abrogated. *Participant reported that cognitive processing was so slow in the upright position that she was “not conversational” until the stimulation was turned on*. AIS-A, American Spinal Injury Association Impairment Scale Grade A; BP, blood pressure; MCA, middle cerebral artery; PCA, posterior cerebral artery; vmean, mean flow velocity; PCAvmean, mean flow velocity; SV, stroke volume; dP/dt, delta pressure over delta time (cardiac contractility); SOL, soleus; TA, tibialis anterior; MH, medial hamstring; VL, vastus lateralis; SCG, superior cervical ganglia; SG, stellate ganglia; CG, celiac ganglia; SMG, superior mesenteric ganglia; IMG, inferior mesenteric ganglia. Color image is available online at www.liebertpub.com/neu

**FIG. 2.**
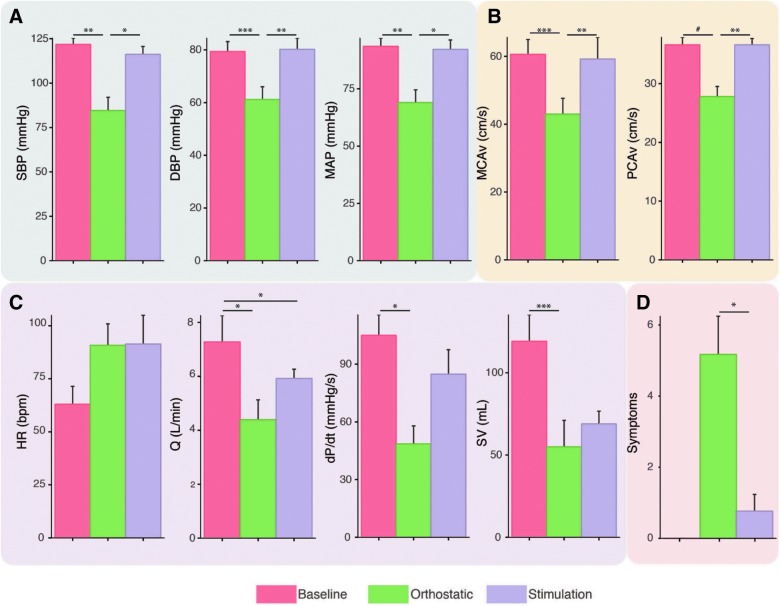
Cardiovascular, cardiac, and cerebrovascular responses to orthostatic challenge were normalized with transcutaneous electrical spinal cord stimulation. (**A**) Blood pressure responses were normalized with stimulation. (**B**) Cerebral blood flow was normalized with stimulation. (**C**) Although systolic function in terms of contractility (dP/dt) was normalized with stimulation, heart rate was still elevated and stroke volume was not restored. (**D**) Symptoms of orthostatic intolerance were almost completely abrogated with stimulation. Repeated-measures ANOVA with Bonferoni contrasts. ^#^*p* = 0.07; *p < 0.05; **p < 0.01; ***p < 0.001. ANOVA, analysis of variance; SBP, systolic blood pressure; DBP, diastolic blood pressure; MAP, mean arterial blood pressure; MCA, middle cerebral artery; PCA, posterior cerebral artery; vmean, mean flow velocity; PCAvmean, mean flow velocity; SV, stroke volume; HR, heart rate; Q, cardiac output; TPR, total peripheral resistance. Color image is available online at www.liebertpub.com/neu

A clinical vignette is presented in Figure 1, demonstrating the integrated cardiovascular and cerebrovascular benefits of transcutaneous spinal cord stimulation. All participants experienced clinically defined OH, which was abrogated with stimulation at the TVII level (Figure 2); however, lower-limb skeletal muscle contraction did not occur, obviating the possibility that the pressor response was attributed to skeletal muscle pump action.^24^ Heart rate did not decrease with stimulation leading SV to still be reduced (Fig. 2).

## Discussion

**FIG. 3.**
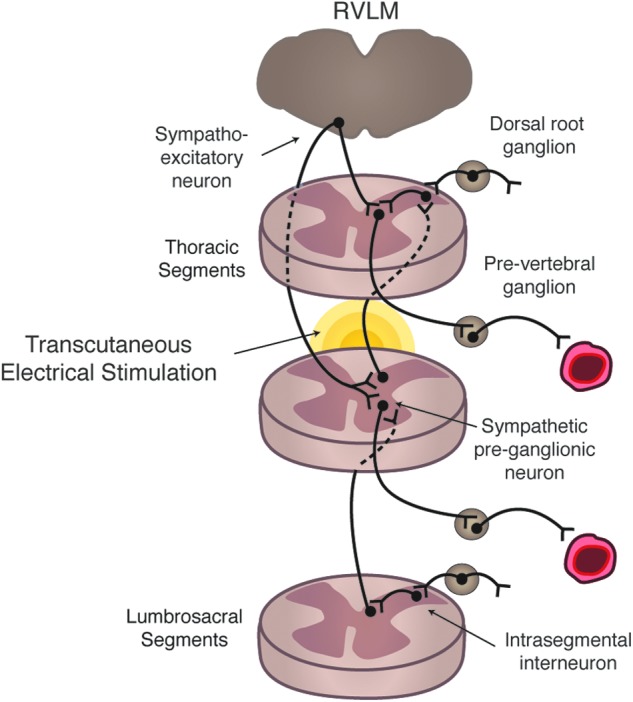
Theoretical framework. Thoracic level stimulation using transcutaneous electrical stimulation excites dorsal afferents that likely excited intersegmental and intrasegmental neurons, which directly and indirectly lead to depolarization of sympathetic pre-ganglionic neurons leading to increased vascular tone. RVLM, rostral ventrolateral medulla. Color image is available online at www.liebertpub.com/neu

Electrical stimulation of the spinal cord, applied transcutaneously, can improve autonomic cardiovascular function after SCI. Specifically, transcutaneous electrical stimulation of the thoracic spinal cord completely abrogated orthostatic hypotension within 60 sec of application. This astonishing and clinically relevant finding was not accompanied by lower-limb skeletal muscle contraction, indicating that the elevations in BP were not attributed to the skeletal muscle pump returning venous blood to the arterial heart, but rather excitation of sympathetic pre-ganglionic neurons leading to vasoconstriction (see Fig. 3). The clinical implications of these realizations are broad and profound, and will be discussed further below.

Crucially, the symptoms of orthostatic intolerance, such as nausea and dizziness, were mitigated or completely abolished with stimulation. An unexpected finding of the present data is that heart rate remained elevated during stimulation despite a restored BP and ensuing baroreflex loading.^18,25^ It may be that stimulation was reaching levels in the spinal cord where cardiac sympathetic pre-ganglionic neurons are located, because we know that the activity of sympathetic interneurons extend much further after SCI.^26^

### Potential mechanisms

Electrical spinal cord stimulation applied noninvasively normalized autonomic cardiovascular dysfunction presenting as OH, as assessed in the present study. After BP, cerebral blood flow, and cardiac function declined drastically with the orthostatic challenge, the stimulation effectively restored these integrated responses to levels observed in the supine position before stimulation. The mechanism through which transcutaneous electrical stimulation elicits integrated cardiovascular benefits was not directly assessed in the present study, and has never been examined. There are at least two possible mechanisms underlying the noted improvements in cardiovascular control. Both of these scenarios depend on sympathetic pre-ganglionic neuron excitation, leading to increased vascular tone, and hence elevations of BP, cerebral perfusion, etc. The first is that primarily small caliber C-fiber afferents may have been excited, which has been reported to elicit uncontrolled exaggerated and often prolonged overactivity of propriospinal interneurons that receive aberrantly elevated sensory input after SCI and lack appropriate supraspinal inhibition to cease excitation once it has occurred (i.e., autonomic dysreflexia^27–30^). The resulting characteristic excitation of pre- and ganglionic neurons, if this was the case, however, would be progressively severe and would result in uncontrolled elevations in BP, and we would expect heart rate to decrease during the dysreflexia period.^7,11,17^ Alternatively, that we observed controlled and stable steady-state increases in BP suggests that propriospinal and sympathetic preganglionic neurons are being excited either directly through the stimulation reaching the spinal cord or by preferential excitation of large diameter sensory axons that do not commonly elicit autonomic dysreflexia.^31^

In support of this, we know that transcutaneous stimulation can recruit the same neural structures as direct epidural stimulation,^32^ which is recognized to heavily involve propriospinal interneurons.^33,34^ Further to this, the elevation in heart rate suggests that the excitatory stimuli is reaching neurons at least at the T4 spinal segments, where cell bodies of cardiac preganglionic neurons are located, which almost certainly requires interneuron excitation.^7,35^ In fact, we know that the excitation of sympathetic preganglionic neurons located several levels rostrally from where the stimulation site is likely, considering that intraspinal sympathetic pathways undergo plastic changes after SCI. Thus, afferent stimuli could enter the spinal cord and reflexively excite preganglionic neurons located a number of spinal segments rostrally.^26^ The specific testing of these mechanisms is outside of the scope of this article.

### Perspectives

The present data may represent the dawning of a new era where autonomic cardiovascular function can be robustly controlled in a safe and tolerable manner. Certainly, the present data suggest that a noninvasive neuroprosthetic device using electrical stimulation to modulate the spinal cord may be a viable new nonpharmacological option to provide a precise and robust strategy to control and/or facilitate effective autonomic function. Its impact could be rapid given the need for new clinical options for managing OH in those living with SCI. In addition, other conditions, such as multiple sclerosis, postural orthostatic tachycardia syndrome, and intramedullary astrocytoma, may benefit with a similar interventional strategy.^7,36^ The potential impact of the implications of the present results is magnified further given that midodrine is considered to be a key front-line pharmacological tool, but the U.S. Food and Drug Administration (FDA) has recently proposed suspending its approval because of lack of clinical evidence.^7^ Recently, droxidopa was approved by the FDA for managing OH; however, because this drug readily crosses the blood–brain barrier, the use of droxidopa in those with altered neurological activity and adrenergic sensitivity could lead to unexpected side effects.^37^ Very recently, a servo-controlled compression device was shown to mitigate the severity of OH, highlighting the interest in utilizing neuroprosthetics as a complimentary therapy to starndard pharmacological interventions for managing this condition.^38^

In the present form, we conservatively chose to limit the duration of the stimulation because of the (remote) possibility of overheating the skin.^39^ The logical next steps are to evaluate the role of more direct spinal cord stimulation either through intraspinal or epidural implants, and to examine whether repetitive electrical stimulation (invasive and noninvasive) of spinal cord circuitry can neurorehabilitate autonomic function, which has been previously suggested.^40^ Another future direction is to determine whether transcutaneous neuromodulation can be used to reduce the severity of transient hypertensive episodes (termed autonomic dysreflexia) after SCI, by modulating the excitability of sympathetic pre-ganglionic neurons.^41^
